# Real-world measurements of ground reaction forces of normal gait of young adults wearing various footwear

**DOI:** 10.1038/s41597-023-01964-z

**Published:** 2023-01-30

**Authors:** Marcin Derlatka, Marek Parfieniuk

**Affiliations:** 1 Bialystok University of Technology, Faculty of Mechanical Engineering, Bialystok, Poland; 2 University of Bialystok, Institute of Computer Science, Bialystok, Poland

**Keywords:** Bone quality and biomechanics, Bone

## Abstract

For years, researchers have been recognizing patterns in gait for purposes of medical diagnostics, rehabilitation, and biometrics. A method for observing gait is to measure ground reaction forces (GRFs) between the foot and solid plate with tension sensors. The presented dataset consists of 13,702 measurements of bipedal GRFs of one step of normal gait of 324 students wearing shoes of various types. Each measurement includes raw digital signals of two force plates. A signal comprises stance-related samples but also preceding and following ones, in which one can observe noise, interferences, and artifacts caused by imperfections of devices and walkway. Such real-world time series can be used to study methods for detecting foot-strike and foot-off events, and for coping with artifacts. For user convenience, processed data are also available, which describe only the stance phase of gait and form ready-to-use patterns suitable for experiments in GRF-based recognition of persons and footwear, and for generating synthetic GRF waveforms. The dataset is accompanied by Matlab and Python programs for organizing and validating data.

## Background & Summary

Clinicians, physiotherapists, and researchers evaluate gait in terms of ground reaction forces (GRFs) between foot and floor. By analyzing GRFs, one can distinguish healthy gait from impaired one^1^, but also can recognize persons and shoes^2,3^.

GRFs are measured by directing a person so as to put a foot on a force plate. It is a solid plate with sensors attached from bottom which convert foot-imposed tensions between plate and floor into electric signals. Alternatively, GRFs can be estimated from other signals, e.g. accelerometer data^4,5^.

GRF-based recognition of persons and shoes, as well as model-based processing of GRFs, are research fields that could develop in the future, as some questions remain open. One problem is that, despite some recent progress^6,7^, no definite methods are known for extracting features related to individual characteristics of a person, not to its temporal conditions. Another challenge is to develop models for synthesizing GRF patterns of a person of a given weight, height, and footwear^5,8–10^. Finally, questions are how to compactly represent and efficiently process large sets of GRF measurements^11^.

In order to tackle these problems, researchers need a lot of data to optimize algorithms, especially via machine learning^12,13^. The research field of GRF analysis had not been well developed in this respect for many years. Majority of publications are based on data of only a dozen or so individuals (see Table 3.1 in^2^), so some results lack strong statistical justification. Moreover, researches commonly used own datasets but had not make them available to the public, so that solutions could not be compared objectively, against the same data.

Just recently, four datasets have been published that could be used to benchmark GRF-related algorithms^7,14–16^.

Following this initiative, we present our collection of 13,702 two-leg measurements of normal-gait GRFs of 324 healthy students. The majority of experiment participants worn footwear of 2 or 7 types, mainly sports shoes, patent leather shoes, and stilettos. So, 754 person-shoe combinations occur in this dataset, each supported by about 18 measurements on average.

To the best of our knowledge, this is the first so-extensive dataset aimed at analyzing GRFs in order to recognize shoe types, and persons regardless of footwear. It could also be interesting to researchers that work on methods for generating synthetic gait patterns and for estimating GRFs from other time series.

But this collection can also be used for purposes different from pattern recognition because it comprises raw, real-world signals, unlike some other publicly available datasets. Moreover, in addition to stance-related samples, preceding and following ones have been provided, in which one can observe noise, interferences, and artifacts caused by imperfections of devices and walkway. Such signals should be welcomed by engineers and scientists interested in developing their own solutions for identifying significant, stance-related samples and for coping with distortions in GRF time series. In particular, unprocessed measurements match the recent trend of pattern recognition based on applying neural networks to raw signals (see e.g.^17^), so as to use machine learning to optimize not only classifiers but also signal transforms for feature extraction. Finally, researches interested in buying force plates, which are expensive, could use this dataset to cheaply familiarize themselves with properties of real-world GRF signals and with issues related to a defective walkway.

Our dataset have already proven to be valuable. Firstly, Derlatka and colleagues used subsets to study methods for GRF-based recognition of shoe types and of persons regardless of footwear^18,19^. Secondly, the measurements were used to develop a novel algorithm for detecting stance-related fragments of signals of the vertical GRF. Thirdly, the data allowed us to invent a method for automatically determining whether left or right foot was put on a particular plate.

The dataset is accompanied by Matlab and Python programs for organizing and validating data. In particular, functions are available that realize the aforementioned innovative algorithms.

For user convenience, processed data have been provided in addition to raw measurements. The former describe only the stance phase of gait and have a character of patterns. So they allow users to easily and quickly, without learning our Matlab and Python programs, set up experiments related to recognizing persons and shoes, and to analyzing GRF signals.

Some of published collections of GRF measurements seem to be similar to our dataset, but they have different properties and applications.

The Gutenberg Gait Database^16^ (GGD) is better suited to analyzing GRFs from the point of view of biomechanics, especially for clinical purposes. It contains mainly data related to barefoot walking and to orthopedic shoes, so it is of limited use for people interested in studying GRF-footwear relations of casual shoes, for non-medical commercial applications. Moreover, its authors claim to publish raw signals, but they have provided only fragments between the foot-strike and foot-off events, and have taken samples not from original signals of a force plate but from their filtered and downsampled versions. Such fragments cannot be used to develop algorithms for gait segmentation, and they lack higher-frequency contents which could be useful for researchers studying nuances of GRFs.

Containing data related to various shoes, the dataset by Duncanson and colleagues^7^ can be used for shoe and person recognition, like our collection. However, it is smaller and less diverse, as many participants tested only one pair or type of footwear, and minority of trials is related to heeled shoes.

## Methods

### Participants

The data have been acquired in a laboratory of the Faculty of Mechanics, the Bialystok University of Technology. Local students voluntarily participated as subjects of gait measurements, while academic staff set up equipment and registered data.

Students were carefully supervised in order to ensure their safety. Before being allowed to the laboratory, they were informed about the purpose and plan of the experiments. Then, each participant was required to sign a written consent. The research had been approved by the Bioethics Commission, Regional Medical Chamber in Bialystok, Poland.

There were 140 (43%) females and 184 (57%) males among the 324 participants. As a result, the dataset comprises 5,949 (43%) and 7,753 (57%) records related to the genders, respectively.

Students were 19–27 years old, with an average age of 21.54 (±1.17) years. The dataset gives some possibilities of analyzing how gait changes with age^7,19^, because 30 students participated in experiments twice, about one year apart.

**Fig. 1:**
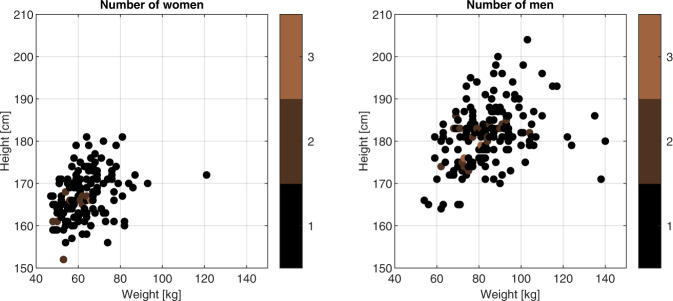
Participants’ weights and heights.

Distributions of students’ weights and heights are shown in Fig. 1. The quantities have been measured for a participant in sports shoes.

No student exhibited noticeable symptoms of abnormal gait. Except for several overweight persons, there were no participant groups that would be easily distinguishable.

### Footwear

The dataset has been created with the aim of obtaining a lot of data that could be used to study algorithms of two classes: (i) methods for GRF-based recognition of persons regardless of footwear, and (ii) solutions for rough yet practical classification of gait with respect of shoe type: soft vs. hard sole, with vs. without heel, and worn by male/female. Such algorithms suit practical applications in which one cannot assume much about footwear worn by people whose gait has to be analyzed.

Because of the aim it was unjustified to use standardized shoes in measurements. But it made sense to roughly standardize footwear so as to diversify it and to facilitate its initial classification. Therefore participants were asked to compare their own sports shoes with other footwear, especially with more formal ones: stilettos and patent leather shoes, in the cases of females and males, respectively.

As few as 44 of 324 students ignored the request and tested only one pair of shoes. The great majority of the participants had responded positively. As many as 250 students tried 2 types of footwear, while 30 participants engaged so much that they used 7 types.

As a result, the dataset comprises 754 person-shoe classes. There are 9 to 27 measurements per class, 18.17 (±2.54) records on average.

Table 1 lists the shoe types that occur in the dataset. It also shows how many measurements and participants are related to each class of footwear. Only sports shoes, stilettos, and patent leather shoes are supported by many records. Nevertheless, to the best of our knowledge, no other published dataset offers a similarly wide range of possibilities of studying effects of footwear on GRF signals.

**Table 1:** Shoe types and numbers of related records and participants.

Code	Shoe type	Number of records	Percentage of records [%]	Number of participants
bal	Ballerinas	304	2.22	16
cnv	Converse-type sneakers	80	0.58	4
flp	Flip-flops	40	0.29	2
hld	Heeled	687	5.01	35
ptn	Patent leather shoes	2996	21.87	180
rnk	Rare (winter boots, trappers, Martens) or unspecified	879	6.42	37
snd	Sandals	221	1.61	11
slp	Slippers	101	0.74	5
snk	Sneakers	156	1.14	8
spr	Sport	6510	47.51	322
stl	Stilettos	1466	10.70	85
wdg	Wedges	262	1.91	15

Shoe types were determined roughly without considering details like sole thickness/stiffness, heel height/diameter, presence/absence of fasteners, shaft height, and others. Neither feet nor shoes were measured, and we did not investigate relations between shoes and feet (tight vs loose, in width/length; slipperiness of socks/insole). It made little sense to care about details in light of our aims mentioned above and thoughts listed below.

It seems impossible to very accurately recognize shoe types when a plethora of various footwear is produced. Firstly, it is unclear how to define fine-grained classes of shoes, when one should consider several dozens of both categorical and continuous features in order to precisely describe a shoe and the shoe-foot relation. Secondly, feature values could occur in numerous combinations, so that one would need rather millions than thousands of measurements to think about discovering subtle relations among shoe details and human gait.

Another problem is that most of shoe details cannot be determined quickly, with the naked eye. Measurement-based in-depth inspection of footwear would considerably lengthen experiments. Students would be discouraged, so that fewer of them would participate, while more participants would test only one pair of shoes. As a result, the dataset would be much less extensive and thus would poorly support statistical analyses and machine learning.

On the contrary, having more data, we could assume that unsupervised classification would reveal new facts about shoe-GRF relations. Such insights could be used to plan further research, in which footwear would be evaluated more precisely.

### Equipment

The data have been acquired by using a pair of portable force plates from the Kistler company (www.kistler.com). One was a 9286A device^20^, the second was the 9286AA model. The former plate used an external 9285A-type charge amplifier, while the second one has an amplifier built-in.

Both force plates have a dimension of 60 × 40 cm. They had been placed on a terracotta floor in midst of a 10-meter walkway constructed of plywood flooring panels.

The devices were arranged in a row, so as to register GRFs of two legs, of one gait cycle. So, we refer to the plates as to the first one and second one, relative to the starting point of participants.

Analog signals of the plates were converted into digital ones by a PCI-6023E I/O card^21^ from the National Instruments. This data acquisition (DAQ) device features 16 channels of analog input. The sampling rate was 960 Hz.

Measurements were registered by using the BTS SMART Capture software executed on a PC workstation. The instruments were carefully inspected, and calibrated if necessary, in accordance with producers’ recommendations, before each experimental session.

Our equipment as well as techniques for data acquisition are similar to those reported by other scientists, please see e.g.^14^.

### Measurement method

Each participant was asked to go cyclically through the walkway for 10 minutes. This time was sufficient to acquire 16–20 correct measurements per participant-shoe class, not being so long to discourage students from participating in the experiment.

Signal registration was manually started and stopped, when a participant was approaching and leaving the plates, respectively. In this way, we were able to register data of one step of gait without many preceding and following, insignificant samples. On average, a GRF measurement comprises 3.66 (±0.61) times more samples than its stance-related fragment. From another point of view, the measurement lasts 2.52 (±0.43) seconds, while the step duration is 0.55 (±0.04) seconds, on average, in this dataset.

Our experiments had to simulate practical situations in which people go through a passage or entrance of limited throughput, possibly controlled by an automatic door or gate. So, participants entered the walkway in response on a voice command, starting from a designated point. They were instructed to shift ahead or behind this point when the previous measurement was unsuccessful, because a foot significantly missed the center of a plate or was put only partially on a plate. Such cases were identified by carefully observing the participant’s feet and by inspecting a data record just after registering it. Unsuccessful measurements were rejected at once, as they would contaminate the dataset, being useless.

Participants were instructed to go in a tempo they consider normal. In addition, the speed of their gait was limited and stabilized only by requiring them to care about the starting point and to rest for 1 minute after every 10 measurements.

Regardless of required breaks, in case of feeling tired or uncomfortable, a participant was allowed to stop and wait for a while, each time before entering the walkway. One could even abandon their participation at any moment, without explanation.

Such an approach was sufficient to obtain measurements that are satisfactorily uniform with respect to gait speed. For participant-shoe classes, the average difference between the shortest and longest durations of steps is only 7.2 (±3) % of the former.

A participant was allowed to put either foot on the first plate. Plate signals can be assigned to legs automatically, by analyzing samples of the medio-lateral GRF. We have developed a novel algorithm for this purpose.

## Data Records

**Fig. 2:**
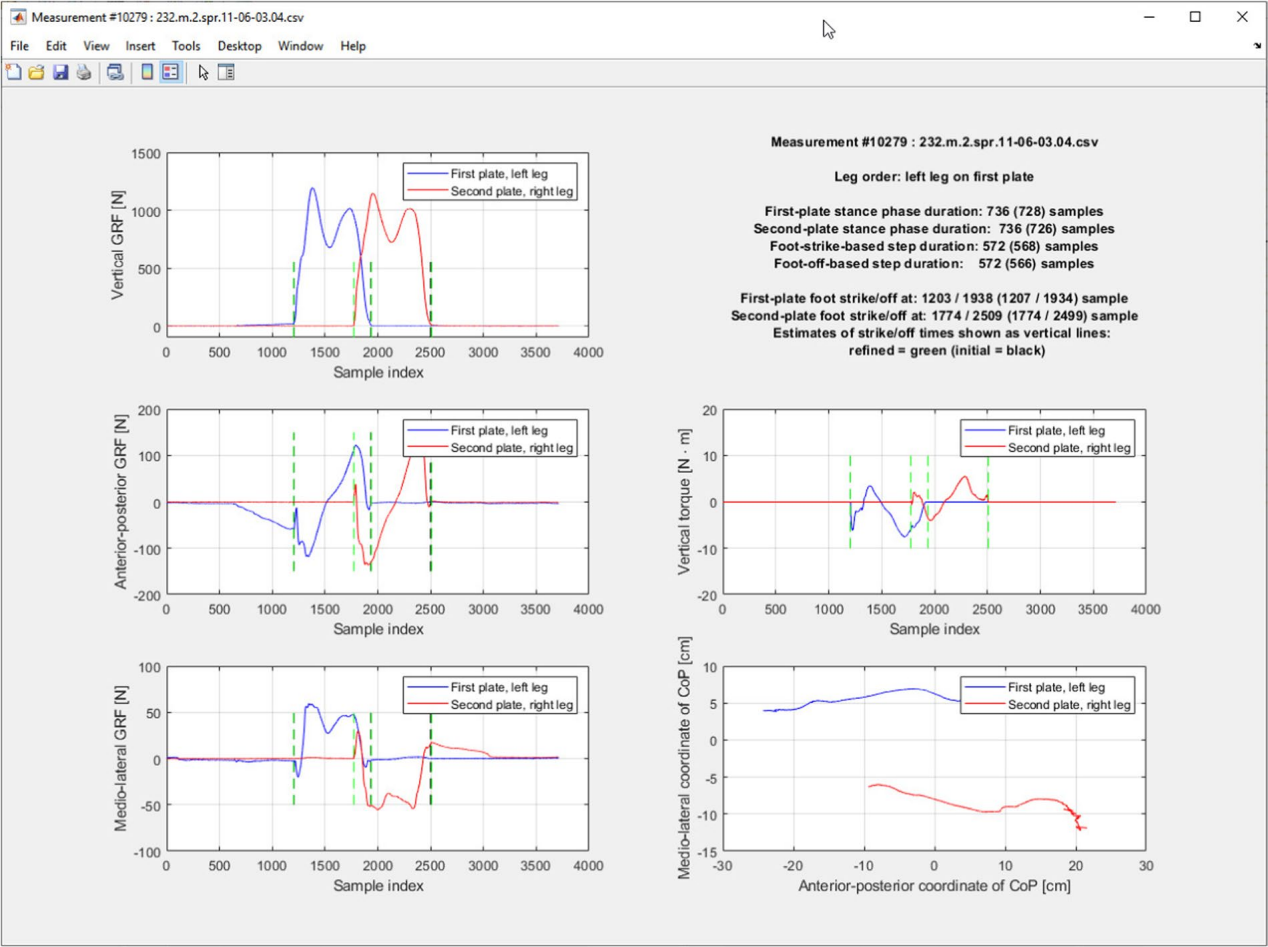
Developed GUI window for inspecting raw data.

Each data record represents one measurement which consists of 12 time series that describe all signals obtainable by default from two force plates. The signals have been listed in Table 2, while their typical waveforms are shown in Fig. 2. Therein, the COP acronym denotes the center of pressure, which can be identified with the force application point.

**Table 2:** Organization of CSV file containing raw data.

Column number	Column identifier	Plate	Signal	Unit
1	firstVForce	First	Vertical GRF	N
2	firstAPForce		Anterior-posterior GRF	N
3	firstMLForce		Medio-lateral GRF	N
4	firstVTorque		Vertical torque	N · m
5	firstAPCoP		Anterior-posterior coordinate of COP	m
6	firstMLCoP		Medio-lateral coordinate of COP	m
7	secondVForce	Second	Vertical GRF	N
8	secondAPForce		Anterior-posterior GRF	N
9	secondMLForce		Medio-lateral GRF	N
10	secondVTorque		Vertical torque	N · m
11	secondAPCoP		Anterior-posterior coordinate of COP	m
12	secondMLCoP		Medio-lateral coordinate of COP	m

An entire record has been written into a single text file in the CSV format, in such a way that samples of a signal form a column. Table 2 shows how signals are associated with columns of a CSV file.

All time series of a measurement comprise as many samples. Series of various records can differ with respect of length, i.e. a file can contain fewer or more lines.

File names are unique, so as to serve as record identifiers. Moreover, they can be used to easily select measurements related to particular participant, gender, and shoe type. This has been achieved by constructing file names in accordance with the following pattern:

ppp.g.s.ttt[−d…d].yy-mm-dd.ffwhere

- ppp: zero-filled index of the participant,
- g: identifier of participant’s gender: ‘f’ for females, and ‘m’ for males,
- s: index of participant’s footwear,
- ttt: identifier of footwear type; in accordance with Table 1,
- [-d…d]: optional, additional description of the footwear; not very meaningful; included into some file names, because it was helpful to us in associating published files with original ones,
- yy-mm-dd: date of the measurement: year (two last digits), month, and day expressed as numbers,
- ff: zero-filled index of the file/record in the participant-shoe class; these indexes do not necessarily form a consistent sequence; missing numbers are indexes of failed, useless measurements, which were not worth publishing.

Each CSV file with raw data has a counterpart whose name is the same but extended with the .PTRN.CSV suffix. The additional file contains processed data listed in Table 3. They describe only the stance-related fragments of the original GRF signals. The fragments have been time-normalized by upsampling so as to contain 1,100 samples between the foot-strike and foot-off events. This round number of samples has been selected to be slightly greater than 1,039, the maximum observed number of stance-related samples. Unlike downsampling, upsampling causes no information loss related to filtering out high-frequency details of signals.

**Table 3:** Organization of CSV file that contains processed data.

Column number	Column identifier	Leg	Force	Data	Unit
1	leftVForce	Left	Vertical	Samples of time-normalized stance-related fragment of signal	N
2	leftAPForce		Anterior-posterior		
3	leftMLForce		Medio-lateral		
4	rightVForce	Right	Vertical		
5	rightAPForce		Anterior-posterior		
6	rightMLForce		Medio-lateral		
7	leftVMean	Left	Vertical	Mean magnitude of 100 pre-foot-strike/post-foot-off raw samples	N
8	leftAPMean		Anterior-posterior		
9	leftAPMean		Medio-lateral		
10	rightVMean	Right	Vertical		
11	rightAPMean		Anterior-posterior		
12	rightMLMean		Medio-lateral		
13	leftRawSampleIndex	Left	All	Index of corresponding raw sample (foot-strike/off-related)	n/a
14	rightRawSampleIndex	Right			

Values are provided for columns 7–14 only in first and last data lines, which describe samples related to foot-strike and foot-off events, respectively.

By only loading contents of PTRN.CSV files into computer memory, without additional processing, a user easily obtains patterns suitable for experiments in GRF-based recognizing shoe types and persons.

Gait data are accompanied by the participantData.csv file which describes all participants: their genders, ages, heights, and weights. Its organization is explained in Table 4.

**Table 4:** Organization of CSV file containing participant data.

Column number	Column identifier	Participant’s feature	Unit/Format
1	index	Index	n/a
2	gender	Gender	‘f’ for females, ‘m’ for females
3	date	Date of measurement	yyyy-mm-dd
4	age	Age	year
5	weight	Weight	kg
6	height	Height	cm
7–10	…2	Date… height of second measurement (Optional)	The same, respectively

The dataset has been deposited in the figshare repository^22^. As there are many data files, they are provided as contents of ZIP archives, in order to allow users to easily download all records and to save disk space. The gaitMeasurementsInCSVFiles.zip archive comprises all CSV files that describe raw measurements, while gaitPatternsInCSVFiles.zip contains all PTRN.CSV files with processed data.

In addition, two MAT-files have been provided in order to allow Matlab users to load data more quickly than from text files. The gaitMeasurements.mat and gaitPatterns.mat files contain the same data as their ZIP counterparts, but measurements have been organized into Matlab variables that combine names and contents of CSV files.

## Technical Validation

**Fig. 3:**
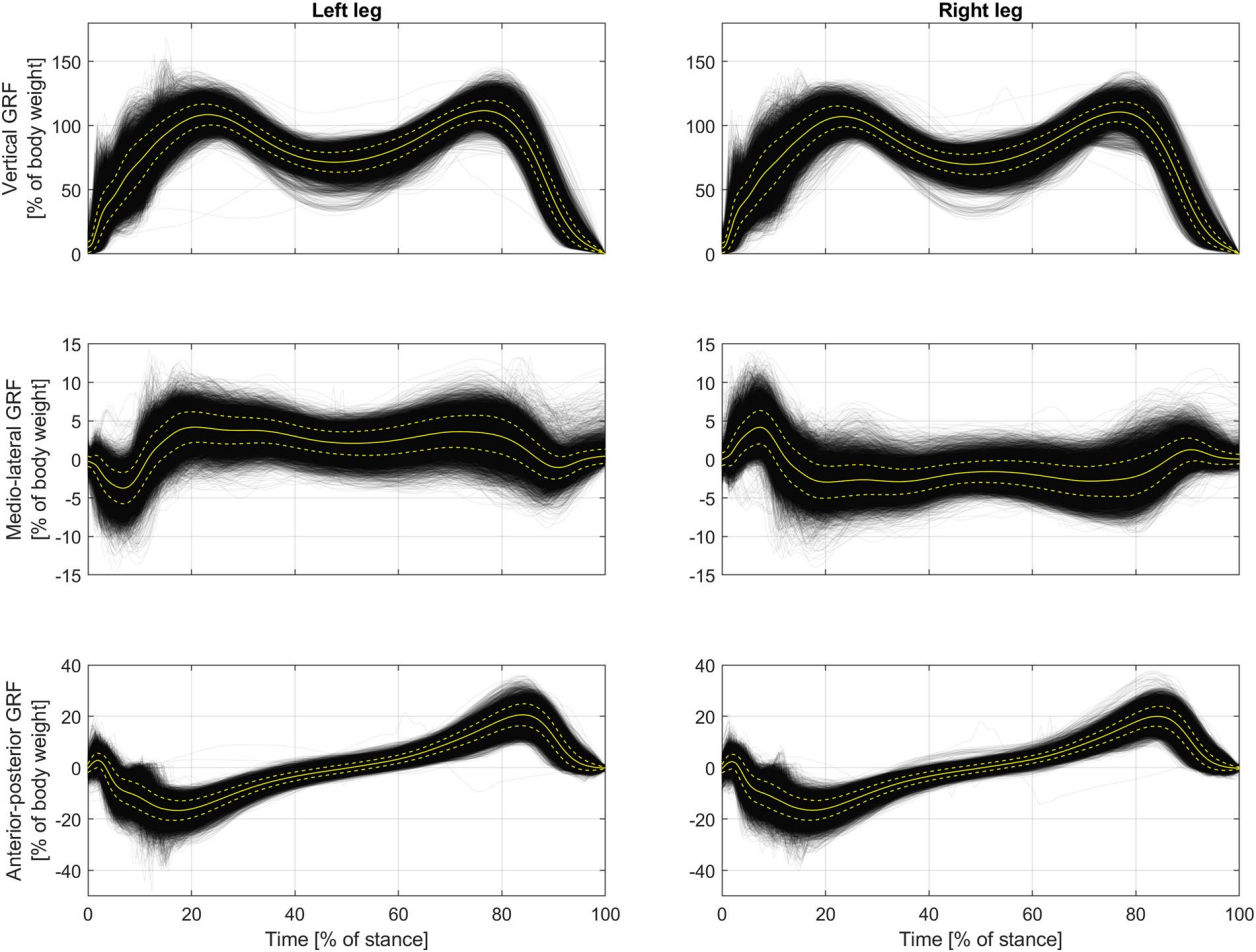
Distributions of samples of weight- and time-normalized versions of stance-related fragments of GRF measurements. Solid and dashed bright lines illustrate means and standard deviations, respectively.

The overall integrity of our data can be assessed by looking at Fig. 3. It illustrates significant, stance-related fragments of GRF signals of all measurements. The fragments, during which a foot contacted a force plate, have been time- and body-weight-normalized, so that a subfigure illustrates the general distribution of stance-related samples related to a particular GRF signal and leg. At the background of a plot cloud, one can see bright curves in solid and dashed lines. These are average curves that illustrate the mean plus/minus standard deviation of a sample distribution.

Figure 3 proves that our measurements are characterized by good quality. Both plot clouds and average curves are consistent with theoretical, ideal waveforms of GRF signals. Dashed-line curves based on standard deviations show that sample distributions are well concentrated around the means. Only a limited number of evident outliers can be noticed, which are easily distinguishable.

Figure 3 is similar to those related to the Gaitrec (see Fig. 3 in^14^) and GGD (see Fig. 2 in^16^) datasets in respect to general curvature, relative positions of characteristic points (main local minimums and maximums), and concentration of sample distributions. Our plots are less smooth, especially at their beginnings. But please notice that Fig. 3 is related to various types of footwear: smoother plots are related to shoes with soft sole, while plots with steps and pulses are related to shoes with hard sole and heels. Overlapping of plots of two kinds results in averages that obviously are less smooth than those related to walking barefoot or in medical shoes, the cases considered by the authors of the previously published datasets. Moreover, in the referenced publications^14,16^ figures are based on low-pass filtered signals, so plots therein obviously are smoother compared to our ones, which are based on signals time-normalized by upsampling.

Our measurements have also been validated by statistically analyzing samples that are related to the stance phase of gait. A useful measurement should contain such samples of both feet, and for each foot, these samples of the vertical GRF should form a consistent wave, which can be reliably distinguished from preceding and following insignificant samples. It is reasonable to assume that if a measurement is correct with respect to the vertical GRF, then it is satisfactory also with respect to the remaining GRF signals.

So, for each measurement we had determined the boundary samples of the stance-related fragments of the vertical GRFs: the maximum sample and those related to foot-strike and foot-off events. Then, indexes and values of these samples were used to compute the correlation between maximum sample and participant weight, ratios among values of boundary samples, duration of the stance phase, step duration, and others. Distributions of there quantities, total and related to participant-shoe classes, were analyzed, by using descriptive statistics and histograms, so as to detect potential outliers. Having noticed an outstanding value, we visually inspected the related measurement.

By publishing histograms in this article, we would make it too lengthy, so they have been printed to a supplementary PDF file, which accompanies the data files at figshare^22^. Alternatively, users can generate histograms by using Matlab/Python programs that accompany the dataset.

Only about 100 (<1%) measurements have been identified that exhibit anomalies in the stance-related wave of the vertical GRF. Outliers are effects of participant obesity, dragging feet, and stumbling. All these are normal for practical measurements of GRFs, and some users would like to see typical outliers and to test methods for data cleaning. So we decided to not artificially over-refine the dataset by rejecting anomalous measurements.

The dataset contains real-world measurements, which suffer from artifacts caused by the equipment. Some issues are typical for sensors and electronic circuits: noise, interference, Gibbs-ringing, and other. Apart from them, a drift-like artifact occurs that is specific for our walkway. We considered a record with artifacts to be acceptable, when they did not prohibit detecting both foot-strike and foot-off events of GRF time series, and had not significantly affect the main features of a stance-related wave: relative times and positions of its main local extrema.

In order to keep this descriptor article clear and brief, we have described properties and causes of the drift-like artifact in a supplementary document that accompanies the dataset at figshare^22^. Herein it seems sufficient to give only the key information.

The drift-like artifact can be noticed in Fig. 2 as non-zero samples that precede the stance-related fragment of the anterior-posterior GRF signal of the first force plate. These non-zero samples rapidly appear and vanish, in consequence of foot-strikes related to measured but also unmeasured gait cycles. A true drift caused by poor calibration of a force plate would have the form of a continuous, constant offset.

The drift-like artifact was caused by faults in the initial construction of the walkway that embraced our force plates. By putting a foot on a walkway element before a plate, a heavier or more energetic participant could bend this element so that its edge touched the force plate. The touch disappeared when another foot contacted the plate, and the body load shifted from the element to that plate. The walkway have been improved after noticing and understanding the problem, before majority of measurements have been acquired. So drift-like artifacts occur in only about 10% of measurements.

The artifact seems considerable compared to stance-related samples and seems to affect many of them. In fact, however, an affected fragment spans only about half of the double support time, starting from the foot strike moment. Neither relative times nor levels of main local extrema are affected, which are used as features for pattern recognition.

So the drift-like artifact does not prevent one from extracting most of the information carried by GRF signals. Only some details about endings of stance-related waves should be considered as uncertain or missing.

As we evaluate records with artifacts to be useful, we have not rejected them from the dataset. But some users could prefer to distinguish them as a separate group, which would be neglected or used only as additional data. Therefore PTRN.CSV files with processed data contain columns that describe, for each GRF of both legs/plates, the mean of the magnitudes of 100 samples that precede the foot-strike event, and the mean of the magnitudes of 100 samples that follow the foot-off event. If a mean is above a threshold, then one may decide that a measurement suffers from unacceptable artifacts.

Histograms of the means confirm that artifacts occur in minority of measurements and only occasionally are large. In particular, the means of the anterior-posterior GRF are lower than 5 N (twice the noise floor) and than 10 N (force plate accuracy, 0.1% of full-scale output) in as many as 9,566 (69.8%) and 12,410 (90.6%) measurements, respectively. It is also notable that the means of vertical GRF are lower than 10 N and than 25 N in as many as 11,868 (86.6%) and 13,580 (99.1%) of measurements.

Please notice that a larger value of 25 N is commonly reported in the literature^14,16^ to be the threshold used to determine stance-related fragments of GRF signals. The idea is to filter a signal of the vertical GRF, and then to search for samples related to threshold crossing^23^. This simple approach was unsatisfactory to us, however.

Results of thresholding highly depend on filtering, while it is difficult to design an optimal filter. A filter should remove both high-frequency noise and low-frequency artifacts like voltage drift. But, the intensity of signal enhancement must be traded off for that of the undesirable side effect of removing useful information^23,24^. The information losses might be related to distorting the general curvature of a GRF waveform and to wiping out impulses caused by hard sole and heels.

So, thresholding tends to signalize foot-strike events later than when they really occurred, while foot-off events are reported too early. Unlike researchers interested in analyzing gait for clinical purposes, we could not accept the risk of detecting a wave of vertical GRF only partially, without transients at its beginning and ending.

Faced with this problem, we have developed a new algorithm for more precisely detecting foot-strike and foot-off events. It is based on analyzing the temporal slope of a signal of the vertical GRF. The method detects a foot-strike by looking for a sample that is preceded by a sequence of almost equal samples and is followed by rapidly increasing values. On contrary, a foot-off is detected as transition from decreasing values to a flat sequence.

We are working on an article describing our algorithm. At the moment, one can conceive its details by studying the Matlab or Python codes that accompany our dataset.

In order to test the algorithm, about 1,000 randomly selected measurements have been reviewed. No problems have been observed in stance-related fragments, which is another proof that our dataset comprises useful signals of good quality.

## Usage Notes

The CSV file format is supported by virtually all professional software for technical and scientific computations, as well as for data analysis. On the other hand, by using popular programming languages, one can rather easily develop a piece of code for reading CSV files. So, interested users should have no problems with loading the dataset into a preferred computer program, commercial or custom.

Instead of unpacking CSV files from a ZIP archive, it is more practical to mount the latter as a virtual disk.

As names of CSV files contain the necessary information, users can easily select records related to a particular participant, gender, and footwear. Regular expressions can be used for this purpose, allowing one to check whether a file name matches a pattern.

The MAT files conform to Version 7.3 of the MAT format, as this version handles large variables and data compression. Matlab has supported such files for many years, since 2006, but they might be unreadable for other computer programs, like Octave, which support only simpler versions of the MAT format.

## Data Availability

The source code of our custom programs in the Matlab and Python languages is publicly available at figshare, under the link to the dataset^22^. These programs allow users to read data from files, to visualize measurements and patterns, to extract samples related to the stance phase of gait, and to perform exploratory analyses of data. The source code has been designed so as to form a programming library, which could be used to develop more advanced applications related to signal processing and pattern recognition. In particular, the Matlab programs are as follows: 1. runMeasurementDemo.m: Demonstrates how to use the remaining programs to access and validate raw measurements and participant data. 2. runPatternDemo.m: Demonstrates how to use the remaining programs to access and validate processed data and participant data. 3. loadParticipants.m: Loads participant data from a CSV file into a structure of arrays. Each array describes one feature of all participants. 4. summarizeParticipants.m: Shows histograms and statistics of distributions of ages, weights, and heights of participants. 5. loadMeasurements.m: Loads raw data of gait measurements from CSV files into an array of structures of vectors. Each structure describes all signals of one measurement. 6. showMeasurement.m: Plots all signals of a given measurement, pointing out their fragments related to the stance phase of gait, between the foot-strike and foot-off events. Creates windows like that in Fig. 2. 7. findStance.m: Determines initial estimates of indexes of raw samples related to the foot-strike and foot-off events. Estimation is based on analyzing the temporal slope of the vertical GRF. 8. cutStance.m: Extracts the stance-related fragment (wave) of a signal of the vertical GRF. Can use extrapolation to refine indexes and values of samples related to the foot-strike and foot-off events, so as to smooth the endings of the fragment. 9. extractStancesFromMeasurements.m: Cuts out samples between the foot-strike and foot-off events from raw GRF signals. 10. summarizeStances.m: Shows histograms, maximums, and minimums of distributions of indexes and values of boundary samples of stance-related fragments of GRF signals. Allows one to identify trends and potential outliers among measurements. 11. showStances.m: Plots stance-related fragments of GRF signals. 12. checkLeftLegFirst.m: Checks whether the first of two series of stance-related samples of the medio-lateral GRF is related to the left leg, while the second is related to the right leg. 13. convertStancesIntoPatterns.m: Time-normalizes stance-related fragments of GRF signals and assigns them to legs. Forms processed data so that they can be directly used as patterns in experiments in recognizing persons and shoes. 14. savePatterns.m: Saves processed data into CSV files. 15. loadPatterns.m: Loads processed data from CSV files into a structure of arrays. Each array describes values of one feature of all files. 16. showPatterns.m: Plots processed data of one or many measurements. Allows one to detect potential outliers among measurements. 17. summarizePatterns.m: Shows histograms, maximums, and minimums related to processed data. Allows one to identify trends and potential outliers. Each file begins with extensive comments that explain its purpose and way of usage. The Python programs have the same names and functionalities as the Matlab ones, and are as well commented, so it would make little sense to list and describe them herein. Only two remarks on them seem to be necessary. Firstly, the programs have been combined into a single module, the gait.py file, so users that would like to develop applications based on our library can import all functions by writing one, simple statement. Secondly, some of our programs show plots in windows, so users should have the Tk GUI back-end installed and associated with the matplotlib library.
